# Autoantibodies in Systemic Lupus Erythematosus Target Mitochondrial RNA

**DOI:** 10.3389/fimmu.2019.01026

**Published:** 2019-05-10

**Authors:** Yann Becker, Geneviève Marcoux, Isabelle Allaeys, Anne-Sophie Julien, Renée-Claude Loignon, Hadrien Benk-Fortin, Emmanuelle Rollet-Labelle, Joyce Rauch, Paul R. Fortin, Eric Boilard

**Affiliations:** ^1^Département de microbiologie et immunologie, Faculté de Médecine de l'Université Laval, Centre de Recherche du CHU de Québec—Université Laval, Québec City, QC, Canada; ^2^Département de mathématiques et statistiques, Université Laval, Québec City, QC, Canada; ^3^Division de Rhumatologie, Département de Médecine, CHU de Québec—Université Laval, Québec City, QC, Canada; ^4^Division of Rheumatology, Department of Medicine, Research Institute of the McGill University Health Centre, Montreal, QC, Canada; ^5^Axe maladies infectieuses et inflammatoires, Centre de Recherche du CHU de Québec—Université Laval, Québec City, QC, Canada

**Keywords:** autoantibodies-blood, mitochondria-RNA, antimitochondrial antibody (AMA), autoimmue disease, systemic lupus erythematosus (SLE), autoantigens, extranuclear nucleic acids

## Abstract

The mitochondrion supplies energy to the cell and regulates apoptosis. Unlike other mammalian organelles, mitochondria are formed by binary fission and cannot be directly produced by the cell. They contain numerous copies of a compact circular genome that encodes RNA molecules and proteins involved in mitochondrial oxidative phosphorylation. Whereas, mitochondrial DNA (mtDNA) activates the innate immune system if present in the cytosol or the extracellular milieu, it is also the target of circulating autoantibodies in systemic lupus erythematosus (SLE). However, it is not known whether mitochondrial RNA is also recognized by autoantibodies in SLE. In the present study, we evaluated the presence of autoantibodies targeting mitochondrial RNA (AmtRNA) in SLE. We quantified AmtRNA in an inducible model of murine SLE. The AmtRNA were also determined in SLE patients and healthy volunteers. AmtRNA titers were measured in both our induced model of murine SLE and in human SLE, and biostatistical analyses were performed to determine whether the presence and/or levels of AmtRNA were associated with clinical features expressed by SLE patients. Both IgG and IgM classes of AmtRNA were increased in SLE patients (*n* = 86) compared to healthy controls (*n* = 30) (*p* < 0.0001 and *p* = 0.0493, respectively). AmtRNA IgG levels correlated with anti-mtDNA-IgG titers (*r*_s_ = 0.54, *p* < 0.0001) as well as with both IgG and IgM against β-2-glycoprotein I (anti-β_2_GPI; *r*_s_ = 0.22, *p* = 0.05), and AmtRNA-IgG antibodies were present at higher levels when patients were positive for autoantibodies to double-stranded-genomic DNA (*p* < 0.0001). AmtRNA-IgG were able to specifically discriminate SLE patients from healthy controls, and were negatively associated with plaque formation (*p* = 0.04) and lupus nephritis (*p* = 0.03). Conversely, AmtRNA-IgM titers correlated with those of anti-β_2_GPI-IgM (*r*_s_ = 0.48, *p* < 0.0001). AmtRNA-IgM were higher when patients were positive for anticardiolipin antibodies (aCL-IgG: *p* = 0.01; aCL-IgM: *p* = 0.002), but AmtRNA-IgM were not associated with any of the clinical manifestations assessed. These findings identify mtRNA as a novel mitochondrial antigen target in SLE, and support the concept that mitochondria may provide an important source of circulating autoantigens in SLE.

## Introduction

The mitochondrion is an intracellular organelle involved in the regulation of numerous cellular functions, among which the best known are ATP production and programmed cell death (1, 2). Mitochondria are considered as deriving from the endosymbiosis of an α-synfular; proteobacterium (3, 4), providing the organelles many bacterial features (3, 5–9).

Different cellular lineages (10–18) may extrude their mitochondria upon activation. Extracellular mitochondria have been identified in damaged tissues (8, 18–20); diverse inflammatory conditions (11, 12, 14, 21–24); and in the blood of critical care patients (22). As mitochondria retained several characteristics of their ancestral prokaryotic origin, the release of mitochondrial components onto the extracellular milieu can activate the innate immune system (25, 26). The efflux of mtDNA is facilitated by megapores formed in the mitochondrial membrane during apoptosis, and detected by the cytosolic DNA sensors cGAS and stimulator of interferon genes (STING) pathway, thereby leading to type I interferon synthesis (27). Cardiolipin, N-formylated peptides, mtDNA, ATP and reactive oxygen species are known mitochondrial damage-associated molecular patterns (9, 28–30). They further activate cells through nuclear oligomerization domain-like receptors (28, 29, 31), toll-like receptors (TLR) (e.g., TLR9 for mtDNA), or formyl peptide receptors (9, 28–31).

**Figure 1:**
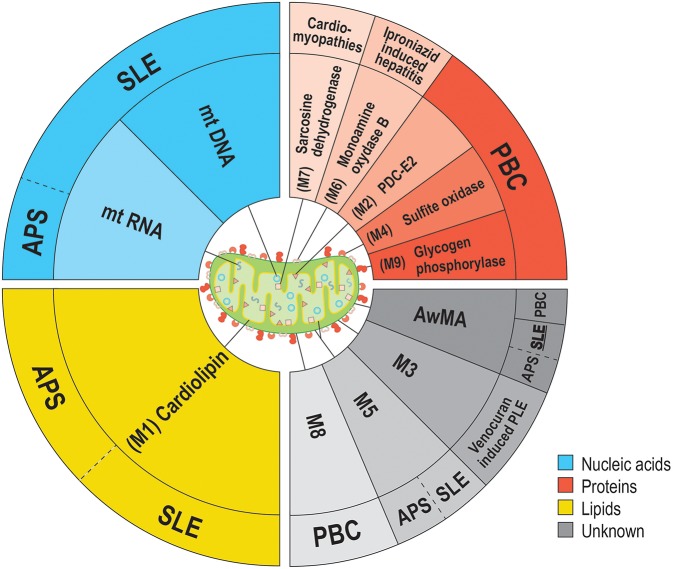
Anti-mitochondrial antibodies and related diseases. Several types of anti-mitochondrial antibodies (AMA) have been reported in various diseases. The epitopes targeted by AMA cover all families of biomolecules: lipids (yellow background), proteins (red hues) or nucleic acids (blue hues). However, the precise nature of some mitochondrial epitopes targeted by AMA are still unclear. (gray hues). To date, the sole mitochondrion-specific phospholipid antigen reported in both APS and SLE is cardiolipin (M1). M1 is located within the mitochondrial inner membrane (MIM) in healthy organelles, but may be displayed on the outer membrane (MOM) upon damages to the organelle. Distinct AMA against an unknown antigen (M5) were also reported in both APS and SLE. Four antigens are associated with PBC; PDC-E2 (M2, MIM), sulfite oxidase (M4, MOM), M8 (MOM), and glycogen phosphorylase (M9, MOM). These mitochondrial antigens are peptidic, with the exception of M8, whose nature remains uncharacterized. Sarcosine dehydrogenase (M7) is another immunogenic protein that is targeted by autoantibodies in patients suffering from cardiac conditions (i.e., hypertrophic or idiopathic cardiomyopathies or acute myocarditis). Two types of AMA were reported as iatrogenically induced in human patients: AMA-M3 (unknown, MOM) and AMA-M6 (monoamine oxidase B, MOM). In addition to these autoantibodies, we have reported the presence of autoantibodies targeting whole mitochondria (AwMA) in patients with SLE, APS, and PBC (with higher titers found in SLE donors). Moreover, antibodies specific to the mtDNA were specific to SLE patients. In the present study, we describe autoantibodies against mtRNA in patients with SLE and APS.

Systemic lupus erythematosus is an autoimmune disease characterized by the presence of circulating immune complexes and inflammation in multiple organs and tissues. Recent evidence point to an involvement of mtDNA, liberated by neutrophils, in the activation of STING and type-I IFN production in SLE (11, 12). Moreover, extracellular mtDNA can enhance leukocyte migration and degranulation (32), and promotes the secretion of the pro-inflammatory cytokine TNF-α by plasmacytoid dendritic cells (33). Production of autoantibodies targeting several mitochondrial components was reported in SLE as well as in other diseases [e.g., primary biliary cirrhosis (PBC), antiphospholipid syndrome (APS), and cardiomyopathies] (Figure 1). Anti-mitochondrial autoantibodies recognize proteins, such as those involved in oxidative phosphorylation, phospholipids or unidentified epitopes present in the mitochondrial membrane. Despite the extensive literature regarding antibodies targeting the cardiolipin (also known as the mitochondrial antigen M1) in SLE, the anti-mitochondrial autoantibody repertoire and their antigenic targets remains mostly uncharacterized (12, 34–37). Using intact mitochondria and mtDNA as antigens to screen autoantibodies in SLE patients, we have shown that different sets of autoantibodies also target the mitochondrial outer membrane and mtDNA (36). Given the accumulating evidence for mitochondrial release during inflammatory pathogenesis, these observations point to a role for mitochondria both in the stimulation of the innate immune system and as a potential source of autoantigens.

Whereas, the mitochondrion has already been described as a source of mtDNA during inflammation (17, 21, 32), it is not known whether its important RNA content (mtRNA) can contribute to the autoantigenic load in SLE. Despite its presence at high copy numbers, the mitochondrial genome is very compact (38–40) During its translation into mitochondrial messenger RNA (38), a long polycistronic transcript is generated from each strand of mtDNA prior to undergoing processing into mtRNA molecules. This highly regulated process is thought to occur in a particular location in the mitochondrion, called mitochondrial RNA granules (41), and requires key RNA processing enzymes such as the members of the FASTK family of proteins (42). The human mitochondrial transcriptome comprises 16S ribosomal RNA molecules (78%), transfer (13%), messenger (8%) and small non-coding antisense (1%) mtRNA molecules. The complete mitochondria transcriptome is controlled by the cell's energy requirements, and therefore varies greatly depending on its tissue distribution. In the heart, 30% of the total messenger RNA molecules are of mitochondrial origin, whereas ~5% of the total messenger RNA load in less metabolically active cells such as leukocytes is encoded by mitochondrial genes (39). The important quantity of mtRNA may thus represent a major antigenic load for the adaptive immune system upon release of mitochondria onto the extracellular milieu.

With the accumulating evidence supporting the liberation of mitochondrial components into the extracellular milieu in SLE (11, 12), it is crucial to identify the various mitochondrial antigens. In the present study, we examined whether the RNA molecules present in mitochondria are antigenic. The levels of anti-mtRNA (AmtRNA) were measured in SLE sera, and we determined whether AmtRNA were associated with antibodies against whole mitochondrial organelles (AwMA) and mtDNA (AmtDNA). We also investigated the occurrence of AmtRNA in an induced model of murine SLE. Finally, we determined whether AmtRNA were associated with disease manifestations in patients with SLE.

## Materials and Methods

### Induced Model of Murine SLE

This study was carried out in accordance with the recommendations of the Canadian Council on Animal Care. The protocol was approved by McGill University Animal Care Committee. C57BL/6 mice were obtained from Harlan Sprague Dawley Inc. (Indianapolis, IN, USA) and housed in a specific-pathogen-free animal facility at the animal facility of the Research Institute of the McGill University Health Center. Female (10–12-weeks-old) mice were injected intravenously (i.v.) with 100 μL human β2-GPI (20 μg) (Crystal Chem Inc., Elk Grove Village, IL, USA), followed 24 h later by a 100 μL i.v. injection of lipopolysaccharide (LPS from *E.coli*, serotype O111:B4; 10 μg) (List Biological Laboratories, Campbell, CA, USA). β2-GPI and LPS injections were repeated every 2 weeks for a total of three rounds of immunizations, and then at 2-month intervals for the fourth and the fifth immunizations. C57BL/6 mice injected i.v. with PBS and LPS following the same schedule were used as controls. Mice were bled 1 week after the fifth immunization and serum was kept frozen at −70°C until testing.

### Mitochondria Isolation

Mitochondria were isolated from the livers of C57BL/6 mice as previously described (43). In brief, cells and tissues were disrupted by grinding in a glass/Teflon tissue potter containing 12 mL ice-cold mitochondrial isolation buffer (10 mM Tris, 1 mM EGTA, 200 mM sucrose) for each gram of liver. Debris were pelleted twice at 700 *g*, for 10 min at 4°C and the supernatants were transferred to fresh tubes. Mitochondria were further separated from other cellular fractions by three centrifugation steps (twice at 7,000 *g* and once at 10,000 *g*, for 10 min at 4°C). Between each step, pelleted mitochondria were re-suspended in 12 mL isolation buffer. Samples were kept at −80°C until required for RNA isolation.

### Mitochondrial RNA Isolation

Mitochondrial RNA was isolated using the Aurum™ Total RNA Mini Kit (Bio-Rad, Hercules, CA, USA), following the manufacturer's instructions. Ribonucleic acid yields were quantified using a BioDrop μLITE and its proprietary software (BioDrop Ltd., Cambridge, UK). The absence of contamination by mitochondrial DNA was assessed by resolution of 1 μg untreated mtRNA and the same amount of RNAse A-treated (QIAgen, 100 μg/mL) mtRNA on a 1.5% (w/v) agarose gel (Supplementary Figure 1A). 15.09 ± 2.74 μg mtRNA were isolated for each mg of bicinchoninic acid assay (BCA)-dosed mitochondria used (*n* = 3).

### Enzyme-Linked Immunoassays for the Detection of Antibodies Targeting Mitochondrial Antigens

Clear 96-well High Bind half-area flat bottom ELISA *microplates* (Corning, New York, USA) were pre-coated with 100 μL per well of 1% protamine sulfate (Sigma-Aldrich) in double-distilled water for 1 h at RT. Plates were then washed thrice with PBS and loaded with mtRNA. Plates were coated overnight at 4°C, washed thrice and non-specific binding was blocked for 4 h at 37°C with 100 μL per well of ELISA blocking buffer (PBS−10% FBS−0.5% gelatin). Wells were rinsed three times with PBS and incubated in duplicate with serum diluted (1:150 for human and 1:50 for mice) in incubation buffer (PBS−10% FCS−0.3% gelatin). Plates were washed thrice with PBS and incubated for 90 min at RT with either γ or μ chain-specific-alkaline phosphatase-(AP) conjugated goat anti-human IgG or IgM (Sigma-Aldrich) for human serum, or γ chain-specific-horseradish peroxidase (HRP)-conjugated goat anti-mouse IgG (Sigma-Aldrich) for mice (1:1000) in secondary antibody buffer (PBS−0.4% bovine serum albumin [BSA]). Unbound antibodies were washed thrice with PBS. Signals from AP-conjugated antibodies were developed with *para*-nitrophenol phosphate (*p*-NPP) for ~30 min at 37°C, and HRP-conjugated antibodies were developed with 3,3′,5,5′-tetramethylbenzidine (TMB) at RT. The reaction was stopped with 2 N sulfuric acid (H_2_SO_4_). Optical densities (OD) were measured at 405 nm (*p*-NPP) or 450 nm (HRP) on a SpectraMax 190 microplate reader (Molecular Devices, Sunnyvale, CA, USA), using SoftMax Pro 5.4.1 (Molecular Devices). For each experiment, blank values (i.e., wells coated with mtRNA, but without sera) were subtracted from each measurement.

The quantity of purified mitochondrial RNA (mtRNA) required for coating half-area flat-bottom 96-well ELISA microplates (Corning, New York, USA) was optimized following the aforementioned protocol, by using increasing concentrations from 0 to 1,600 ng of coating mtRNA. Pooled sera (1:150) from 6 SLE patients, who had previously tested positive for AmtDNA and AwMA, were incubated after blocking non-specific binding. The peak signal for optical densities at 405 nm was obtained with 200 ng of coating mtRNA (Supplementary Figure 1B).

### Ethics and Study Approval

This study was carried out in accordance with the recommendations of the Research Ethics Board of the CHU de Québec—Université Laval with written informed consent from all subjects. All subjects gave written informed consent in accordance with the Declaration of Helsinki. The protocol was approved by Research Ethics Board of the CHU de Québec—Université Laval.

### Human Serum Samples

The human sera tested in this study were obtained from the Systemic Autoimmune Rheumatic Disease (SARD) biobank and data repository (SARD-BDB) located at the *CHU de Québec-Université Laval* (UL). This SARD-BDB and the specific use of the sera for the present study were approved by the *CHU de Québec-UL* research ethics board (#B13-06-1243 and #B14-08-2108, respectively). Patients with SLE met the 1982 ACR classification criteria for SLE (revised in 1997) (44, 45). A peripheral blood sample was collected at the time of their first visit. Serum samples from 30 healthy donors and 87 SLE patients included in the SARD-DBD cohort were used in the present study. However, one patient had no clinical data available and was therefore excluded for bio-statistical comparisons (i.e., *n* = 86 SLE donors for these tests).

### Additional Serum Samples

Sera from a cohort of patients and controls from the University of Toronto Lupus Clinic, as well as patients with primary biliary cirrhosis (PBC) from Quebec City, were used in additional exploratory analyses to test the presence of AmtRNA in patients with the antiphospholipid syndrome (APS, *n* = 12) and PBC (*n* = 12). APS patients and healthy controls, distinct from those included in the SARD-BDB (*n* = 43), were originally recruited between August 2010 and October 2011, and gave consent to allow remaining biospecimens to be used for future studies on lupus biomarkers. This study has been reviewed and approved by the Research Ethics Board of the University Health Network (#10-0637-BE) and of the CHU de Québec—Université Laval (#B14-08-2108). APS patients met 1999 Sapporo criteria for the disease (revised in 2006) (46, 47), and healthy controls were recruited if they had no known illnesses and had no infectious symptoms at the time of the blood draw. Donors gave a single blood sample that was linked to their anonymized clinical data. PBC patients were positive for anti-mitochondrial antibodies and presented clinical criteria for the disease (47, 48).

### Clinical Variables Collected in SLE Patients

#### Sociodemographic Variables

Information was collected concerning patient's age, gender, marital status, and ethnicity at the first visit in the SARD-BDB.

#### Patient Characteristics Including Exposures to Cardiovascular Risk Factors

A body mass index (BMI) was calculated and reported as underweight, normal, overweight and obese. Hypertension and diabetes mellitus were documented as present or absent. Smoking history was reported as non-smokers, ex-smokers or current smokers. Female patients were considered post-menopausal in the absence of menstruations for more than 12 continuous months.

#### Disease Specific Characteristics

ACR classification criteria (44, 45) were documented for each of the 11 categories and a total score calculated (5 ± 1.28). Disease duration, lupus disease activity using the SLE Disease Activity Index–2000 (SLEDAI-2K) (49, 50) and lupus damage using the Systemic Lupus International Collaborating Clinics (SLICC)/ACR damage index (SDI) (51, 52) were collected during the clinical visit matched to the blood specimen draw. Both the SLEDAI-2K and the SDI are reported as continuous variables and they both have proven validity, reliability, and perform well in observational studies.

#### Medication Variables

Antimalarial use was defined as use of hydroxychloroquine or chloroquine at the current visit. Steroid use was defined as prednisone use in the past year.

#### Clinical Outcomes

Clinically relevant lupus disease activity and damage were used as clinical outcome in our analyses and were defined as a SLEDAI-2K of 4 or more to capture clinically active lupus and a SDI of 1 or more to capture clinically significant damage. Other outcome variables included arterial and venous thrombotic event ever in the past and presence of lupus nephritis according to the presence or absence of the renal item of the SLICC Classification criteria for SLE (53). Presence or absence of carotid plaques, as well as average carotid-intima media thickness (CIMT) was also documented by carotid ultrasound following a standard examination of both carotids (standard carotid ultrasound research protocol using an Esaote MyLab Five ultrasound machine with digital images sent for blind reading at the IMT Core Laboratory of the Montreal Heart Institute).

#### Information From Clinical Laboratories

For SLE patients, an automated complete blood count was documented. The anti-dsDNA, anticardiolipin antibodies (aCL) (IgG and IgM—laboratory cut-offs of 40 GPL or MPL units) and anti-β2-GPI (IgG and IgM—laboratory cut-offs above the 99th percentile of controls) were measured by ELISA. The lupus anticoagulant assay (LA) followed international guidelines for the performance of this functional assay (54). The above tests were performed in a clinical laboratory at CHU de Quebec-Universite Laval as part of routine care.

#### Information From Research Laboratories

In addition to the measurements provided by the clinical laboratories, our research laboratory performed antibody assays to detect AwMA and AmtDNA, following previously described methods (36).

### Statistical Analyses

Descriptive statistics are presented as mean with standard deviation or frequency with percentage without missing values for continuous and categorical variables, respectively. Comparisons between groups were performed using the Student's, Wilcoxon or Kruskal-Wallis tests depending on the nature of the variables and their distribution. Spearman correlations were calculated to assess association between continuous variables. Associations between AmtRNA and clinical outcomes were studied by bivariate and multivariate logistic regressions, for dichotomous and continuous outcomes, respectively. The latter were adjusted for gender, disease duration, age, BMI, antimalarial medication and prednisone use. ROC curves were generated to assess the predictive ability of AmtRNA to discriminate between SLE and controls, and their area under the curve (AUC) was calculated. Participants' results were considered positive for AmtRNA when their value was above the cut-off value identified after maximizing Youden's Index. A 95% confidence interval was obtained for the cut-off using 10,000 bootstrap samples. Performance measures are presented with their 95% exact confidence interval. Statistical analyses were performed with Prism 7 software (GraphPad Software Inc., La Jolla, CA, USA) and SAS version 9.4 (SAS Institute Inc., Cary, NC, USA) and Figures were assembled with Photoshop CS6 13.0 (Adobe Systems Inc., Mountain View, CA, USA).

## Results

**Figure 2:**
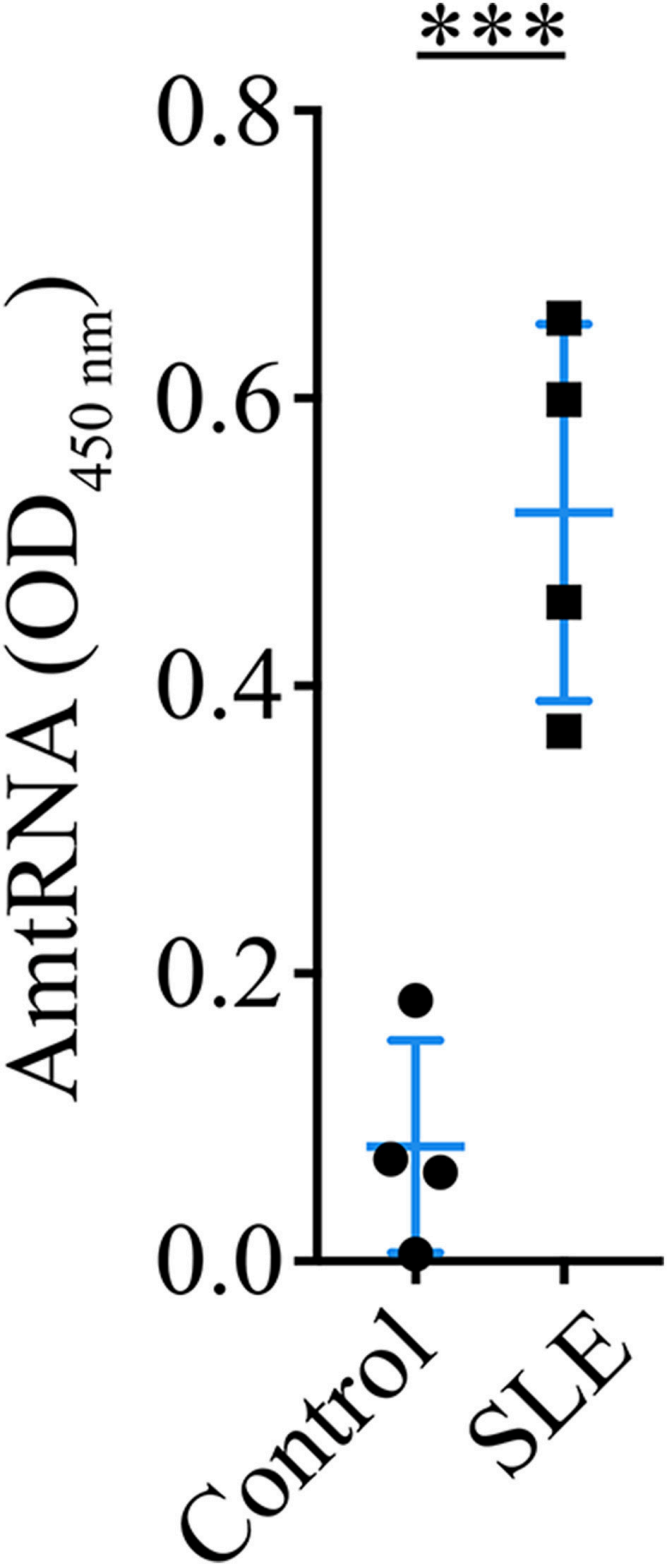
Circulating anti-mitochondrial RNA autoantibodies are detectable in sera from mice with induced SLE. Sera (1:50) from mice with induced SLE were incubated on ELISA plates coated with 200 ng murine mtRNA per well. Mice with induced SLE displayed a significant increase in serum antibodies against mtRNA in comparison to control mice. *N* = 4 mice per group. Data show the mean ± SD. Student's *t*-test. ^***^p < 0.001.

We used our quantitative ELISA to assess whether AmtRNA from the IgG subclass (AmtRNA-IgG) could be detected in an induced model of murine SLE in which the production of circulating IgG against whole mitochondria (AwMA) and mitochondrial DNA (AmtDNA) was previously reported (36). Antibodies against mtRNA were significantly increased (*p* = 0.0005) in the sera of SLE mice compared with control mice (Figure 2).

**Figure 3:**
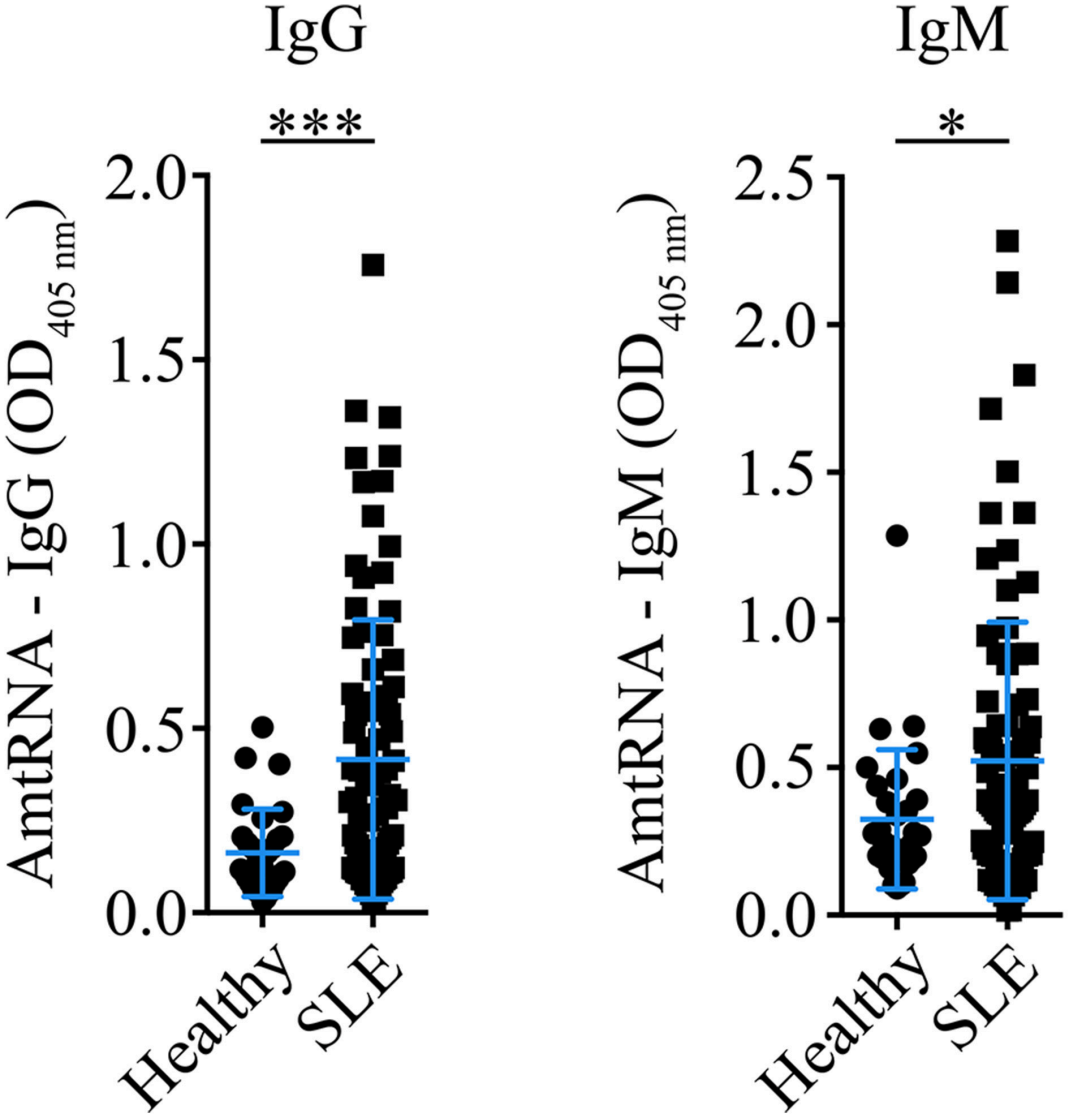
Antibodies targeting mitochondrial RNA (AmtRNA) are elevated in SLE patients. Two different isotypes of antibodies against mtRNA, IgG (**left** panel) and IgM (**right** panel), were assessed in SLE patients and healthy individuals included in the SARD-BDB. Both AmtRNA IgG and IgM were significantly increased in SLE patients, compared to healthy individuals (*p* = 0.0002 and *p* = 0.0493, respectively). SLE: *N* = 86; Healthy controls: *N* = 30. Data show the mean ± SD. Student's *t*-test. ^*^p < 0.05; ^***^p < 0.001.

A cohort of 86 SLE patients (Tables 1–5) and 30 healthy controls (19 females [63.3 %], 11 males [36.7%], age: 49.33 ± 7.68 years) was studied to determine the occurrence of AmtRNA-IgG and AmtRNA-IgM in human SLE. The proportion of male donors in the healthy group was higher than in the SLE cohort (i.e., 36.7% vs. 16.3% of male donors, respectively) as well as than the 1:10 male-to-female sex bias reported in the disease. We thus verified that the anti-mitochondrial antibody titers measured were not influenced by sex, using Wilcoxon test and found no significant differences (*p*-values between 0.14 and 0.97). Both AmtRNA-IgG and -IgM were significantly increased in SLE patients, compared with healthy individuals (*p* = 0.0002 and *p* = 0.0493, respectively) (Figure 3; Supplementary Figure 1). In healthy donors, AmtRNA-IgM were higher than AmtRNA-IgG levels (0.32 ± 0.24 vs. 0.16 ± 0.12), suggesting that antibodies targeting mitochondrial epitopes may be present in healthy individuals even in the absence of any detectable pathology.

**Table 1:** Sociodemographic characteristics in the SARD-BDB.

**Variable**	***n***	**Mean ± SD [or *n* (%)]**
Female	86	72 (83.7)
Age (years)	86	49.41 ± 14.60
Marital status		
Single	82	14 (17.1)
Married		55 (67.1)
Tobacco intake		
Non-smokers	83	48 (57.8)
Smokers		14 (16.9)
Ex-smokers		21 (25.3)

**Table 2:** Clinical characteristics in the SARD-BDB.

**Variable**	***n***	**Mean ± SD [or *n* (%)]**
Disease duration	86	10.43 ± 10.69
Body mass index	86	25.55 ± 4.97
Post-menopausal	64	38 (59.4)
Hypertension	86	11 (12.8)
Diabetes	84	2 (2.4)
Malar rash	85	19 (22.4)
Discoid rash	85	12 (14.1)
Photosensitivity	85	36 (42.4)
Oral ulcers	85	26 (30.6)
Arthritis (≥2 peripheral joints)	85	69 (81.2)
Serositis	85	22 (25.9)
Renal disorders	85	22 (25.9)
Neurological disorders	85	4 (4.7)
Hematological disorders	85	68 (80.0)
Immunological disorders	85	62 (72.9)
Anti-nuclear antibodies (ANA)	85	85 (100.0)

**Table 3:** Outcome variables of the study.

**Variable**	***n***	**Mean ± SD [or *n* (%)]**
SLEDAI-2K (Score)		3.24 ± 3.96
SLEDAI-2K ≥ 4	86	36 (41.9)
SDI (score)		3.24 ± 3.96
SDI ≥ 0	86	36 (41.9)
Thrombosis		10 (11.6)
Arterial events	86	3 (3.5)
Venous events		4 (4.7)
Presence of plaque in the carotid	63	24 (38.1)
Carotid intima-media thickness (CIMT, μm)	34	0.63 ± 0.13
Nephritis	61	14 (23.0)

*SDI, lupus severity disease index; SLEDAI-2K, systemic lupus erythematosus disease activity index−2000*.

**Table 4:** Information about medications taken by SLE patients (*n* = 86) in the SARD-BDB.

**Variable**	***n*(%)**
Anticoagulation/anti-platelets	13 (15.1)
Antimalarial	70 (81.4)
Prednisone	18 (20.9)
Lipid lowering	14 (16.3)
Diabetes medication	2 (2.3)
**LUPUS TREATMENTS**	
Hydroxychloroquine	65 (76)
Chloroquine	6 (7)
Azathioprine	15 (17)
Methotrexate	15 (17)
Leflunomide	1 (1)
Mycophenolate mofetil	11 (13)
Mycophenolic acid	1 (1)
Cyclophosphamide (PO or IV)	3 (4)

*IV: intravenous injection; PO: per os*.

**Table 5:** Laboratory measurements.

**Variable**	***n***	**Mean ± SD [or *n* (%)]**
Platelets (0.10^∧^9/L)	86	221.63 ± 72.68
White blood cells (0.10^∧^9/L)	86	5.80 ± 2.06
Creatinine clearance	26	91.88 ± 21.31
AmtDNA (OD 405 nm)		
IgG	86	0.49 ± 0.53
IgM	86	0.45 ± 0.38
AwMA (OD 405 nm)		
IgG	86	0.34 ± 0.37
IgM	86	0.56 ± 0.58
AmtRNA (OD 405 nm)		
IgG	86	0.42 ± 0.38
IgM	86	0.52 ± 0.47
Lupus anticoagulant (LA)	61	8.69 ± 22.76
Anticardiolipin antibodies (aCL)		
IgG	79	11.33 ± 12.39
IgM	79	6.92 ± 13.92
Anti-β2GPI antibodies		
IgG	79	2.78 ± 6.59
IgM	79	3.36 ± 3.89
Anti-dsDNA antibodies	22	31.01 ± 80.40

*β_2_GPI, β-2-glycoprotein I; AmtDNA, anti-mitochondrial DNA antibodies; AmtRNA, anti-mitochondrial RNA antibodies; AwMA, anti-whole mitochondria antibodies; OD, optical density*.

**Figure 4:**
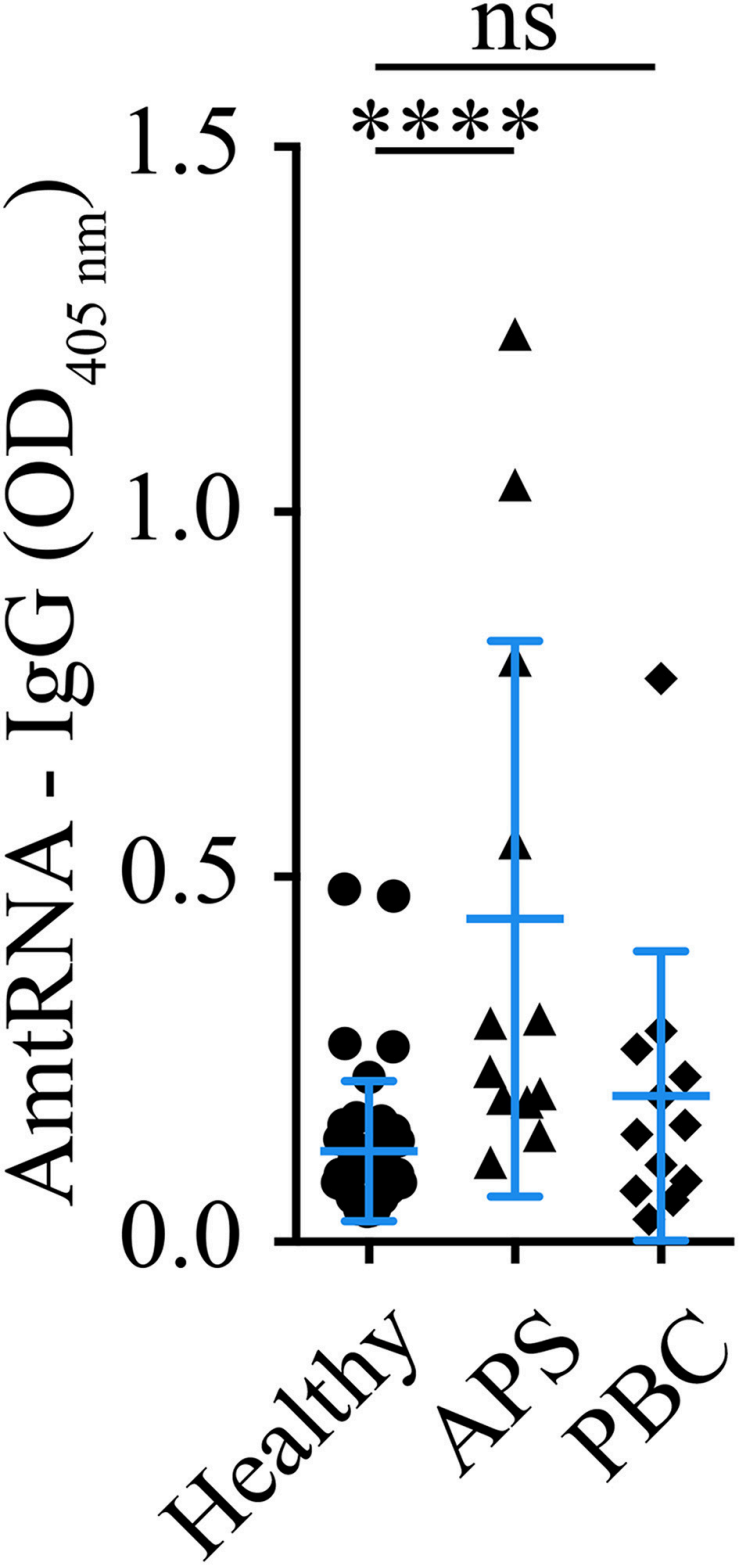
Detection of AmtRNA in two different diseases with anti-mitochondrial antibodies. Antiphospholipid syndrome (APS) and primary biliary cirrhosis (PBS) are two diseases with antibodies targeting mitochondrial antigens; cardiolipin (M1) in APS and PDC-E2 (M2), sulfite oxidase (M4), M8 (whose target is still unclear) and sarcosine dehydrogenase (M9) in PBC. Sera (1:150) from patients with APS presented a significant increase in circulating autoantibodies against mtRNA, compared to healthy individuals, whereas PBC patients had levels similar to the controls. Healthy: *N* = 43, APS: *N* = 12, PBC *N* = 12. Data are Mean ± SD. Kruskal-Wallis test with multiple comparisons to controls/healthy donors; Dunn's correction. ^****^p < 0.001.

In a separate exploratory analysis using donors distinct from those included in the SARD-BDB, AmtRNA-IgG were also significantly increased in patients with APS, an autoimmune condition often associated with SLE (*p* < 0.001 vs. healthy controls). However, no differences in AmtRNA-IgG were observed between patients with PBC, a disease known for an adaptive immune response against mitochondrial autoantigens, and healthy controls (*p* = 0.31) (Figure 4).

Autoantibodies to genomic dsDNA (anti-dsDNA) and to β-2-glycoprotein I (anti-β_2_GPI, IgG, and IgM) were evaluated during the clinical work-up of a patient with an increased likelihood of SLE. We examined whether titers in AmtRNA (IgG and IgM) and levels of anti-dsDNA or anti-β_2_GPI were associated with each other in the patients, and found correlations between levels of AmtRNA-IgG and those of both anti-β_2_GPI-IgG and IgM (*r*_s_ = 0.22, *p* = 0.05). AmtRNA-IgM titers only displayed a strong correlation with anti-β_2_GPI-IgM (*r*_s_ = 0.48, *p* < 0.0001). Conversely, no correlations were observed between AmtRNA and concentrations of anti-dsDNA (Table 6). We also determined whether the levels of AmtRNA correlated with IgG and IgM antibodies targeting mitochondrial epitopes localized in diverse sub-compartments of the organelle (36). Specifically, we measured antibodies recognizing intact whole mitochondria (AwMA), which most likely bind epitopes found on the outer mitochondrial membrane; aCL, which target cardiolipin, a phospholipid located mainly within the mitochondrial inner membrane; and AmtDNA, which recognize mitochondrial DNA. We found that AmtRNA-IgG levels correlated with AmtDNA-IgG (*r*_s_ = 0.54, *p* < 0.0001) and with AwMA-IgG (*r*_s_ = 0.24, *p* = 0.03), but not with aCL (IgG and IgM). AmtRNA-IgM concentrations correlated with AmtDNA-IgM (*r*_s_ = 0.83, *p* < 0.0001), AwMA-IgM (*r*_s_ = 0.71, *p* < 0.0001), aCL-IgG (*r*_s_ = 0.27, *p* = 0.02), and aCL-IgM (*r*_s_ = 0.57, *p* < 0.0001). Thus, in addition to the newly described AmtRNA, different sets of anti-mitochondrial antibodies occur conjointly in SLE.

**Table 6:** Correlations of anti-mtRNA levels, with titers of other auto-antibodies in SLE patients.

		**AmtRNA**	
		**IgG**	**IgM**
AmtDNA	IgG	***r***_**s**_ **=** **0.54**	*r*_s_ = 0.19
		***p*** **<** **0.0001**	*p* = 0.08
	IgM	*r*_s_ = −0.01	***r***_**s**_ **=** **0.83**
		*p* = 0.92	***p*** **<** **0.0001**
AwMA	IgG	***r***_**s**_ **=** **0.24**	*r*_s_ = 0.14
		***p*** **=** **0.03**	*p* = 0.21
	IgM	*r*_s_ = −0.03	***r***_**s**_ **=** **0.71**
		*p* = 0.78	***p*** **<** **0.0001**
AmtRNA	IgG	/	*r*_s_ = 0.16 *p* = 0.15
	IgM	*r*_s_ = 0.16	/
		*p* = 0.15	
Anti-β_2_GPI antibodies	IgG	***r***_**s**_ **=** **0.22**	*r*_s_ = 0.18
		***p*** **=** **0.05**	*p* = 0.11
	IgM	***r***_**s**_ **=** **0.22**	***r***_**s**_ **=** **0.48**
		***p*** **=** **0.05**	***p*** **<** **0.0001**
Anti-dsDNA antibodies		*r*_s_ = 0.13	*r*_s_ = 0.11
		*p* = 0.56	*p* = 0.62

*Values are presented as Spearman correlation coefficient (r_s_) and p-value*.

*β_2_GPI, β-2-glycoprotein I; aCL, anti-cardiolipin antibodies; AwMA, anti-whole mitochondria antibodies. AmtDNA, anti-mitochondrial DNA antibodies; Anti-dsDNA, antibodies against double-stranded DNA. Data in bold are statistically significant (p < 0.05)*.

One of the main features of SLE is the expression of numerous autoantibodies in patients (55), some of which are known to be associated with the clinical expression of the disease (56). We assessed whether AmtRNA are qualitatively associated with positivity to several autoantibodies commonly found in SLE, including anti-dsDNA, aCL, and LA. AmtRNA-IgG levels were higher in presence of anti-dsDNA antibodies (*p* < 0.0001), whereas AmtRNA-IgM titers were elevated in presence of aCL-IgG and -IgM (*p* = 0.01 and *p* = 0.002, respectively) (Table 7). Of note, circulating AmtRNA-IgM tended (*p* = 0.06) to be increased in the presence of LA in SLE patients.

**Table 7:** Association of AmtRNA with clinically relevant SLE autoantibodies.

		**AmtRNA**	
		**IgG**	**IgM**
aCL	IgG	(–) 0.26 ± 0.43	**(**–**) 0.34** **±** **0.40**
		(+) 0.42 ± 0.91	**(+) 0.56** **±** **0.90**
		*p* = 0.14	***p*** **=** **0.01**
	IgM	(–) 0.26 ± 0.43	**(**–**) 0.33** **±** **0.38**
		(+) 0.53 ± 0.95	**(+) 0.86** **±** **1.43**
		*p* = 0.19	***p*** **=** **0.002**
Lupus anticoagulant		(–) 0.25 ± 0.45	(–) 0.35 ± 0.40
		(+) 0.40 ± 0.67	(+) 0.57 ± 0.98
		*p* = 0.20	*p* = 0.06
Anti-dsDNA antibodies		**(**–**) 0.19** **±** **0.28**	(–) 0.34 ± 0.41
		**(+) 0.70** **±** **0.71**	(+) 0.55 ± 0.27
		***p*** **<** **0.0001**	*p* = 0.10

*Values presented as median ± IQR and Wilcoxon test p-value for patient positives (+) or negatives (–) for each variable*.

*aCL, anti-cardiolipin antibodies; AmtRNA, anti-mitochondrial RNA antibodies; Anti-dsDNA, antibodies against double-stranded DNA. Data in bold are statistically significant (p < 0.05)*.

We examined whether AmtRNA were associated with disease manifestations in 86 SLE patients for whom detailed clinical information were available (Table 8). Higher levels of AmtRNA-IgG were associated with a lower occurrence of plaque in the carotid using a bivariate analysis [OR(95% CI) = 0.14 (0.02–0.91); *p* = 0.04], but this significance was lost in the multivariate logistic regression [OR(95% CI) = 0.16 (0.01–1.81); *p* = 0.14]. We found no association between AmtRNA-IgG and two clinical indices; one measuring SLE disease activity (SLEDAI-2K ≥ 4) and the other indicating damages (SDI > 0), both by bi- and multivariate analyses. However, higher concentrations of AmtRNA-IgG were positively associated with elevated anti-dsDNA antibodies in both models. AmtRNA-IgG were not associated with lupus nephritis in a bivariate analysis [OR(95% CI) = 0.17 (0.02–1.71); *p* = 0.13], but this association became significant in the multivariate model [OR(95% CI) = 0.02 (0.00–0.68); *p* = 0.03]. In contrast, AmtRNA-IgM were not significantly associated with any of these clinical outcomes by either the bi- or multi-variate analysis.

**Table 8:** Association of AmtRNA with clinical manifestations in SLE.

	**AmtRNA**			
	**IgG**		**IgM**	
	**OR (CI)**	***p***	**OR (CI)**	***p***
Thrombotic events	1.28 (0.24;6.77)	0.77	0.93 (0.22;3.93)	0.92
	[1.15 (0.17;7.87)]	[0.88]	[1.00 (0.18;5.61)]	[1.00]
Presence of plaque	**0.14 (0.02–0.91)**	**0.04**	0.83 (0.25–2.76)	0.76
	[0.16 (0.01–1.81)]	[0.14]	[0.82 (0.23–2.91)]	[0.76]
SLEDAI-2K ≥ 4	2.30 (0.73–7.26)	0.16	0.86 (0.34–2.17)	0.75
	[3.04 (0.78–11.77)]	[0.11]	[0.68 (0.25–1.88)]	[0.46]
SDI ≥ 0	0.95 (0.28–3.21)	0.94	0.50 (0.16–1.58)	0.24
	[0.85 (0.15–4.92)]	[0.85]	[0.46 (0.11–1.86)]	[0.28]
Positivity to anti-dsDNA antibodies	**34.97 (6.26–195.55)**	**<0.0001**	1.92 (0.67–5.50)	0.23
	**[70.60 (6.31–789.47)]**	**[0.0005]**	[2.01 (0.50–8.11)]	[0.33]
Lupus nephritis	0.17 (0.02–1.71)	0.13	0.43 (0.08–2.30)	0.33
	**[0.02 (0.00–0.68)]**	**[0.03]**	[0.25 (0.04–1.48)]	[0.12]

*Values presented as odds ratios (95% Wald Confidence Interval) and p-value from logistic regressions. In each instance, bivariate results are followed by multivariate analysis (between square brackets). Values in bold have a p-value ≤ 0.05*.

*AmtRNA, anti-mitochondrial RNA antibodies; CI: 95% Wald Confidence Interval; OR, odds ratio; SDI, lupus severity disease index; SLEDAI-2K, SLE disease activity index−2000. Data in bold are statistically significant (p < 0.05)*.

Furthermore, we assessed if our conclusions were identical in patients with higher disease activity by repeating our calculations with patients having a SLEDAI-2K score > 6 (i.e., for 15 patients, compared to 36 with a cut-off value at a SLEDAI-2K score ≥ 4). The associations between AmtRNA-IgG with SLEDAI-2K > 6 were [OR(95% CI) = 2.71 (0.71–10.31)] for the bivariate logistic regression and [OR(95% CI) = 1.99 (0.40–10.00)] for the multivariate regression model. Values for the associations between AmtRNA-IgM and SLEDAI-2K > 6 for bi- and multivariate analyses were [OR(95% CI) = 0.53 (0.12–2.25)] and [OR(95% CI) = 0.37 (0.07–1.89)], respectively. Thus, the conclusions remain the same using either SLEDAI-2K cut-off value.

To determine whether AmtRNAs might qualify as efficient predictors of SLE, we optimized cut-off values by Youden's method (Table 9). Calculated cut-off values were 0.30 for AmtRNA-IgG and 0.52 for AmtRNA-IgM. Both parameters were very specific for SLE (0.90 for IgG and 0.87 for IgM). Even though both Ig isotypes displayed a certain lack of sensitivity [43 SLE patients (49%) positive for AmtRNA-IgG and 33 (38%) for IgM], their positive predictive values (0.93 and 0.89) suggest that AmtRNAs may be considered as biomarkers of interest. Importantly, of all of the anti-mitochondrial autoantibodies measured, AmtRNA-IgG was the most potent at discriminating SLE patients from healthy donors. In this regard, AmtRNA-IgG was closely followed by AmtDNA-IgM. In contrast, AwMA (IgG and IgM) and AmtDNA-IgG failed to efficiently discriminate SLE patients from healthy controls.

**Table 9:** Performance of cut-off values for AmtRNA, AwMA, and AmtDNA (OD 405_nm_).

		**Cutpoint (95% BCI)**	**Sensitivity (95% ECI)**	**Specificity (95% ECI)**	**PPV (95% ECI)**	**NPV (95% ECI)**	**AUC (95% ECI)**
AmtRNA	IgG	**0.30 (0.11–0.54)**	**0.49 (0.38–0.60)**	**0.90 (0.73–0.98)**	**0.93 (0.82–0.99)**	**0.38 (0.27–0.50)**	**0.72 (0.62–0.82)**
	IgM	**0.52 (0.24–0.64)**	**0.38 (0.28–0.49)**	**0.87 (0.69–0.96)**	**0.89 (0.75–0.97)**	**0.33 (0.23–0.44)**	**0.62 (0.51–0.72)**
AwMA	IgG	0.30 (0.12**–**0.44)	0.36 (0.26**–**0.47)	0.80 (0.61**–**0.92)	0.84 (0.68**–**0.94)	0.30 (0.21**–**0.42)	0.57 (0.45**–**0.69)
	IgM	0.68 (0.19**–**1.37)	0.24 (0.16**–**0.35)	0.87 (0.69**–**0.96)	0.84 (0.64**–**0.96)	0.29 (0.20**–**0.39)	0.48 (0.37**–**0.60)
AmtDNA	IgG	0.44 (0.22**–**1.25)	0.35 (0.25**–**0.46)	0.77 (0.58**–**0.90)	0.81 (0.65**–**0.92)	0.29 (0.19**–**0.40)	0.51 (0.40**–**0.62)
	IgM	**0.36 (0.24–0.57)**	**0.51 (0.40–0.62)**	**0.83 (0.65–0.94)**	**0.90 (0.78–0.97)**	**0.37 (0.26–0.50)**	**0.65 (0.55–0.75)**

*Values in bold have an AUC significantly different than 50%*.

AmtDNA, anti-mitochondrial DNA antibodies. AmtRNA, anti-mitochondrial RNA antibodies. AUC, area under the curve. AwMA, anti-whole mitochondria antibodies. BCI, Bootstrap Confidence Interval. ECI, Exact Confidence Interval. NPV, Negative Predictive Value. OD, optical density. PPV, Positive Predictive Value. Data in bold are statistically significant (p < 0.05)

## Discussion

Although the interplay between extracellular mitochondria and innate immunity has been well-described, the interactions between mitochondria and the adaptive immune system are less appreciated. Mitochondrial components are generally seen as potential damage-associated molecular pattern (DAMP) if released by cells, but their inflammatory potential may be different if they are also recognized by autoantibodies. Herein, we propose mtRNA as a novel source of mitochondrial autoantigens with high relevance to SLE.

Mitochondrial RNA is not the only mitochondrial sub-component with antigenic potential in SLE. The first descriptions of anti-mitochondrial antibodies (AMA) were published in the 1980's. However, the actual epitope(s) of some AMA remain unidentified (57). Thus, AmtRNA add to the more recently appreciated AmtDNA and AwMA (36). Adaptive autoimmunity targeting mitochondrial motifs is not unique to SLE: a humoral immune response against mitochondrial autoantigens was reported in various diseases, and described as 9 different types of AMA targeting distinct epitopes (namely, M1 to M9) (36, 57). While AMA have been observed in different contexts such as in cardiovascular diseases, iatrogenic disorders, secondary syphilis, APS and SLE, they are best characterized in PBC (57, 58). The latter is characterized by progressive infiltration of autoreactive lymphocytes through the hepatic portal system (48, 59, 60). These cells display targeted autoreactivities directed against different mitochondrial antigens specifically expressed by bile ducts (60) such as the E2 subunit of the pyruvate dehydrogenase complex (PDC-E2, also known as mitochondrial antigen M2) (61–64), sulfite oxidase (M4) (65), glycogen oxidase (M9) (47, 66, 67) as well as other antigens that have not yet been described (M8). Detection of these AMA in PBC by ELISA have a prognostic value: patients positive for AMA-M4 and -M8 suffer from active and/or progressive forms of the disease (68), whereas patients with only AMA-M2 and -M9 display diseases with delayed evolutions (69) (recapitulated in Figure 1).

How exactly the mitochondrial antibodies are produced is not completely understood, but mitochondrial antigens can be generated through the degradation of old or damaged mitochondria by a specific form of autophagy known as mitophagy. Autophagosomes containing mitochondria travel through the endolysosomal system, leading to the degradation of its cargo and allowing the production of mitochondrial peptides that can be processed and expressed by the major histocompatibility complex (MHC). Both MHC-I and MHC-II have been implicated (70), an involvement for the latter being suggested in the surveillance of mitochondrial mutations occurring in cancer (71, 72). However, a recent study revealed that mitochondrial antigen processing can also occur independently of mitophagy. In this case, mitochondrial antigens are carried to endosomes by mitochondrial-derived vesicles formed by a mechanism regulated by the proteins PINK1 and Parkin (73, 74). Whether these mechanisms are involved in the processing of mtRNA molecules remains to be established.

Mitochondrial RNA is a recognized trigger of TLR8, which similarly to bacterial RNA, stimulates peripheral blood mononuclear cells (75). As the most metabolically active cells express more mtRNA, they are more likely to contribute to mtRNA antigenic load (39). Our study demonstrates that mtRNA is also recognized by antibodies, suggesting that Fc receptors may be implicated in the internalization of mtRNA-IgG complexes by endosomes, thereby favoring interactions with TLR8. Mitochondria express various RNA species, the main one being ribosomal 16S RNA molecules (39). However, the respective antigenicity of each mtRNA species was not assessed in the present study. Moreover, the presence of certain nuclear messenger RNA has been described within mitochondria (39), which could also account for the antigenicity potential of the mitochondria. Considering the evidence for mitochondrial release in different pathogeneses, our demonstration of the presence of antibodies directed against mitochondrial RNA further confirms the role of mitochondria as a source of autoantigens in autoimmunity.

We observed associations between the three sets of mitochondrial antibodies (AwMA, AmtDNA, and AmtRNA), pointing to their common source. Moreover, both AmtDNA-IgG and AmtRNA-IgG were associated with positivity for anti-dsDNA antibodies, suggesting close relationships between auto-antibodies targeting distinct nucleic acids.

While the IgG targeting mtRNA were significantly elevated in SLE patients, the IgG recognizing mtDNA and whole mitochondria were not increased in these patients. These observations contrast with our previous findings, which involved a different cohort of patients and showed that AmtDNA and AwMA were significantly increased in SLE (36). The patients included in our previous work were recruited by the University of Toronto Lupus Clinic. The patients recruited in the present study (SARD-BDB) are characterized by a shorter median duration of the disease (10 vs. 6 years) that may account for reduced organ damage as indicated by the SDI score (median: Toronto = 1; SARD-BDB = 0) and the frequency of patients with lupus nephritis (Toronto: 38.5%; SARD-BDB: 16.3%). These differences may reflect the course of the disease with earlier titers of autoantibodies clearing detrimental circulating autoantigens (i.e., in the SARD-BDB cohort) until other pathophysiological processes such as epitope spread occur, eliciting immune complex-mediated organ damage. Another interesting aspect is the discrepancy between the representation of the various ethnicities included in both cohorts. The SARD-BDB is almost exclusively composed of Caucasians (Caucasian: 97.7%, Black: 1.2%, Other ethnicities: 1.2%), whereas the Toronto cohort includes a more diverse ethnic panel (Caucasian: 57 %, Black: 18 %, Asian: 21%. Other: 5%). Such differences between two groups of patients may also impact results such as the incidence, prevalence and mortality rates (76–79). Together, these differences may reflect upon the protective effects of AmtRNA-IgG reported in the present study. Moreover, these elements suggest that the spectrum of anti-mitochondrial antibodies may shift during the course of the disease.

The heterogeneity of the disease duration for the SLE patients included in the SARD-BDB allows the optimization of cut-offs by Youden's method that discriminate positive from negative samples. However, calculation of a universal cut-off requires detection of AmtRNA in newly-diagnosed SLE patients. Additionally, associations between AmtRNA and clinical features of the disease should be interpreted with caution, as clinical outcomes identified might have occurred before or at the same time than the blood draw. Verification of the temporal relationship between the production of AmtRNA and clinical outcomes would require a study of the variation in anti-mitochondrial antibodies titers over time and their levels at the onset of a clinical outcome in a large prospective inception cohort.

Systemic lupus erythematosus is a highly complex disease, many aspects of which still elude researchers (80). To date, only a limited number of biomarkers are available (81, 82). There is an intense effort to discover new biomarkers that would allow specific discrimination of SLE patients from both healthy individuals and those with diseases that have clinical features close to those of SLE (83). From this perspective, we present AmtRNA-IgG as antibodies present in SLE and APS, two diseases that are often associated with each other. Interestingly, AmtRNA-IgG appeared to be associated with less lupus nephritis and plaque formation in the carotid. Together, these elements indicate that AmtRNA may have prognostic value and help to identify patients with specific clinical profiles. Moreover, the different associations of AmtDNA and AmtRNA with lupus nephritis (AmtDNA are positively associated with nephritis, while AmtRNA display a negative association) may help predict SLE patients at risk of kidney damage.

Our study highlights that expression of a broad repertoire of anti-mitochondrial antibody subtypes (AMA; AMA-M1, AMA-M5, AwMA, AmtDNA, AmtRNA) is a major feature of SLE, with specific targets being associated with different clinical features. Future studies dedicated to the characterization of the mitochondrial autoantigens recognized in SLE and their outcome on disease progression may provide useful information that will ultimately help to improve diagnosis, prognosis, and stratification of SLE patients.

## Ethics Statement

This study was carried out in accordance with the recommendations of the Research Ethics Board of the CHU de Québec—Université Laval with written informed consent from all subjects. All subjects gave written informed consent in accordance with the Declaration of Helsinki. The protocol was approved by Research Ethics Board of the CHU de Québec—Université Laval.

## Author Contributions

Experiments were conceived and designed by YB, IA, PF, and EB. JR contributed critical reagent, resources, and expertise. Experiments were performed by YB, GM, IA, and HB-F. Data were processed and analyzed by YB, GM, IA, R-CL, A-SJ, and supervised by R-CL, A-SJ, PF, and EB. The manuscript was written by YB, GM, PF, and EB, and critically reviewed by all authors.

### Conflict of Interest Statement

The authors declare that the research was conducted in the absence of any commercial or financial relationships that could be construed as a potential conflict of interest.

## Abbreviations

β_2_GPI: β-2-glycoprotein I
aCL: anticardiolipin antibodies
AMA: antimitochondrial antibodies
AwMA: anti-whole mitochondria antibodies
LA: lupus anticoagulant
mtDNA: mitochondrial DNA
mtRNA: mitochondrial RNA
SARD-BDB: systemic auto-immune rheumatic disease biobank and data repository.
