# Supplementary material for: Autoantibodies in Systemic Lupus Erythematosus Target Mitochondrial RNA
Source: Front Immunol. 2019 May 10;10:1026. doi: 10.3389/fimmu.2019.01026 (PMC6524553; doi:10.3389/fimmu.2019.01026)
Supplement: Supplementary file 1 [file Data_Sheet_1.docx]

**Autoantibodies in systemic lupus erythematosus target Mitochondrial RNA**

Yann Becker^1^, Geneviève Marcoux^1^, Isabelle Allaeys^1^, Anne-Sophie Julien^2^, Renée-Claude Loignon^3^, Hadrien Benk-Fortin^1^, Emmanuelle Rollet-Labelle^1^, Joyce Rauch^4^, Paul R. Fortin^1,3,5^*, Eric Boilard^1,5^*.

^1^ Centre de Recherche du CHU de Québec – Université Laval, Département de microbiologie et immunologie, Faculté de Médecine de l’Université Laval, Québec, Qc, Canada.

^2^ Département de mathématiques et statistique, Université Laval, Québec, Qc, Canada.

^3^ Division de Rhumatologie, Département de Médecine, CHU de Québec – Université Laval, Québec, Qc, Canada.

^4^ Division of Rheumatology, Department of Medicine, Research Institute of the McGill University Health Centre, Montreal, Qc H4A 3J1, Canada

^5^Axe maladies infectieuses et inflammatoires, Centre de recherche du CHU de Québec – Université Laval, Québec, Qc, Canada.

To whom correspondence should be addressed:

Eric Boilard, PhD

Centre de Recherche du CHU de Québec – Université Laval

2705 Laurier, room T1-49

Québec, G1V 4G2

Québec, Canada

E-mail: [eric.boilard@crchudequebec.ulaval.ca](mailto:eric.boilard@crchudequebec.ulaval.ca)

And

Paul R. Fortin, MD, MPH, FRCPC

Centre de Recherche du CHU de Québec – Université Laval,

2705 boulevard Laurier, room TR-93,

Quebec City, Quebec, G1V 4G2, Canada.

Telephone (418) 654-2178

facsimile (418) 654-2142;

E-mail: [paul.fortin@crchudequebec.ulaval.ca](mailto:paul.fortin@crchudequebec.ulaval.ca)

**Supplementary Figure:**


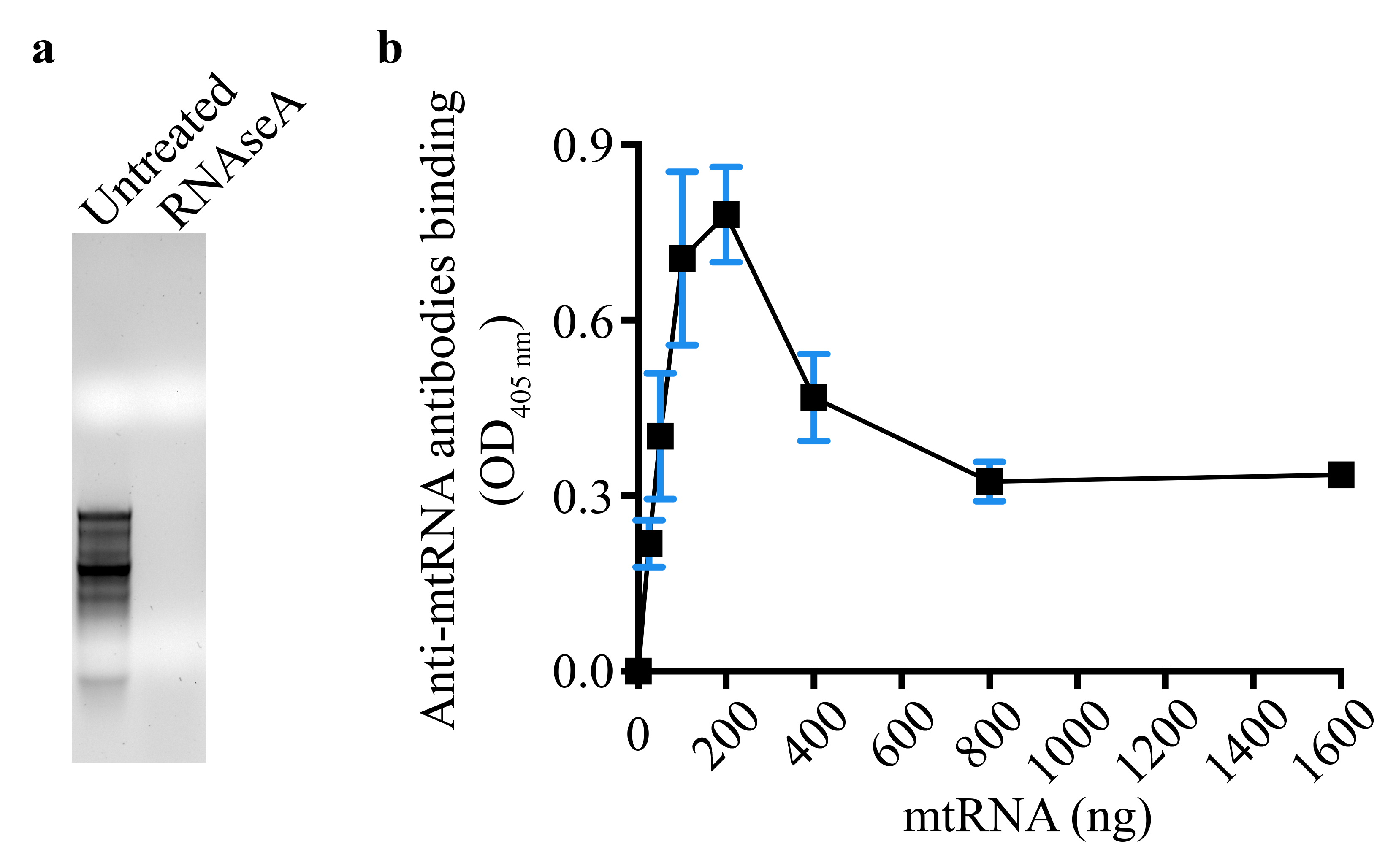


**Supplementary Figure 1: Saturation of ELISA microplates by mitochondrial RNA.**

(a) Mitochondrial RNA (mtRNA) was isolated from C57BL/6J mice. For quality control, mtRNA treated or not with RNAseA were resolved on an 1.5% agarose gel. Full-length image representative of 3 different experiments. (b) Increasing concentrations of mtRNA (0-1600 ng, n = 3) were used to cover protamine sulfate pre-treated ELISA microplates. After blocking of non-specific binding and incubation with pooled sera from 6 SLE patients, plates were incubated with secondary antibodies specific to γ chains of human IgG coupled with alkaline phosphatase. Signal was revealed using *para*-Nitrophenolphosphate (p-NPP) and read on a microplate reader. The maximum signal was obtained using 200 ng mtRNA. Data are Mean ± SD.
